# Exploring the paradox between perception and actual childhood drowning risk: an evidence-based study in Bangladesh

**DOI:** 10.3389/fped.2026.1843830

**Published:** 2026-07-02

**Authors:** Edris Alam, Md Mostafizur Rahman, Tannin Jeffrey, Denboy Kudejira

**Affiliations:** 1 Faculty of Resilience, Rabdan Academy, Abu Dhabi, United Arab Emirates; 2 Department of Geography and Environmental Studies, University of Chittagong, Chittagong, Bangladesh; 3 Department of Disaster Management & Resilience, Faculty of Arts and Social Sciences, Bangladesh University of Professionals, Dhaka, Bangladesh; 4 Disaster and Development Organization (DADO), Chattogram, Bangladesh; 5 African Synthesis Centre for Climate Change, Environment and Development (ASCEND), University of Cape Town (UCT), Cape Town, South Africa

**Keywords:** children, drowning, perception, public health, risk, safety

## Abstract

Childhood drowning is a major public health issue in Bangladesh, especially in rural and island regions. This cross-sectional study explored the gap between perceptions and actual drowning risks by surveying 322 respondents from the northern mainland and Sandwip Island. A purposive sampling strategy was used to identify households with child drowning experiences, and data were collected using a culturally adapted, semi-structured questionnaire. Statistical analyses, including logistic and linear regression, examined associations between perceptions, drowning mortality, and sociodemographic factors. Although 90.37% of respondents believe drowning is preventable, only 5.28% teach their children how to respond during incidents, and 84.47% lack rescue knowledge. Surprisingly, willingness to send children to childcare centers was associated with higher reported drowning deaths (aOR: 3.69). Geographic and income disparities influenced risk perception, with island residents reporting lower awareness (*β* = −0.04) and higher-income respondents showing greater perception. Findings highlight a critical mismatch between awareness and action, emphasizing the need for community-based prevention strategies, safety audits of childcare facilities, and targeted interventions for low-income populations.

## Introduction

Drowning continues to be an under-recognized epidemic that claims the lives of thousands of children worldwide each year. It is the third leading cause of unintentional injury-related mortality worldwide and accounts for approximately 9% of all injury-related fatalities (1). Low- and middle-income nations experience a disproportionately high number of drownings (1–5). Among these countries, Bangladesh experiences a particularly high burden of childhood drowning, especially among young children living in rural and water-abundant environments where supervision, safety infrastructure, and access to preventive interventions remain limited (6–9).

Alarmingly, drowning is the primary cause of death for children aged 1–4 years in numerous countries, reflecting the disproportionate vulnerability of this age group (8). It is estimated that over 90% of drowning fatalities occur in low- and middle-income countries, revealing stark global inequalities in exposure, supervision, infrastructure, and access to preventive interventions (1). The global burden of childhood drowning extends well beyond mortality, imposing profound psychological and emotional consequences on affected families (10), social and economic burdens on vulnerable communities (11), and additional pressure on already overstretched public health systems in low- and middle-income countries (5). As a largely preventable cause of death and injury, childhood drowning represents a critical public health challenge that demands immediate, sustained, and coordinated action across policy, health, and community-based interventions.

Childhood drowning does not occur only in natural water bodies such as ponds, rivers, canals, and wetlands. A considerable number of drowning incidents among infants and toddlers also occur within or around the home environment, including household ponds, water storage containers, bathtubs, buckets, and swimming pools, particularly where supervision is inadequate (1, 12, 13). Young children are especially vulnerable because even small amounts of standing water can pose fatal risks. In many low- and middle-income settings, household water storage practices and unrestricted access to bathrooms or nearby water sources increase the likelihood of unintentional drowning among toddlers. These findings emphasize the importance of integrating household-level water safety measures, close supervision, and early preventive education into broader childhood drowning prevention strategies.

Childhood drowning remains a significant public health concern in Bangladesh, with water bodies posing constant risks in many communities. The situation is particularly dire in Bangladesh, which records among the highest drowning rates globally. An estimated 18,268 children under the age of 18 die from drowning each year, equivalent to approximately 50 deaths per day (14). Vulnerability is unevenly distributed, with children living in rural areas facing a 4.5-fold higher risk of drowning compared to their urban counterparts (9). This elevated risk is exacerbated by the country's geographical characteristics, including many rivers, wetlands, and frequent annual monsoons.

Despite the scale and severity of the problem, childhood drowning in Bangladesh remains a relatively neglected public health issue, necessitating immediate attention from public health professionals, policymakers, and researchers (11). Pilot drowning-prevention interventions implemented in parts of central and southern Bangladesh have generated important insights into patterns of risk. These studies identify several factors associated with both fatal and non-fatal drowning, including, being male, being under five years old, maternal illiteracy, seasonal variation, and lower socioeconomic status (7, 9, 12).

Building on this emerging evidence base, child drowning prevention efforts in Bangladesh have evolved considerably in recent years. Early initiatives, such as the community-based “crèches” intervention, demonstrated positive outcomes by providing supervised spaces for young children, thereby reducing drowning risk (15). This approach has since been scaled up through the Integrated Community-Based Childcare (ICBC) program, extending coverage to a wider range of communities and strengthening child supervision in high-risk areas. Nevertheless, significant gaps remain, as drowning continues to pose a critical challenge, particularly in northern districts that have yet to be fully incorporated into the ICBC program's scope.

Despite the growing programmatic efforts, there remains a notable gap in empirical research on how childhood drowning is perceived by local communities across different regions of Bangladesh.

However, limited attention has been paid to northern and island regions, where geographical and social contexts may influence both exposure and responses to drowning risk. Understanding local perceptions is important, as this shapes risk awareness, supervision practices, and community engagement with prevention initiatives.

The objective of this study is to examine perceptions of childhood drowning in the northern region and island areas of Bangladesh, with particular attention to how these perceptions vary across sociodemographic characteristics. Generating such evidence is essential for informing more effective and targeted drowning-prevention policies, enabling better prioritization of limited resources, and supporting the design of culturally appropriate and context-specific interventions. Beyond Bangladesh, these insights may also contribute to drowning-prevention strategies in other low- and middle-income countries facing similar structural and environmental challenges.

## Materials and methods

### Study design

**Figure 1:**
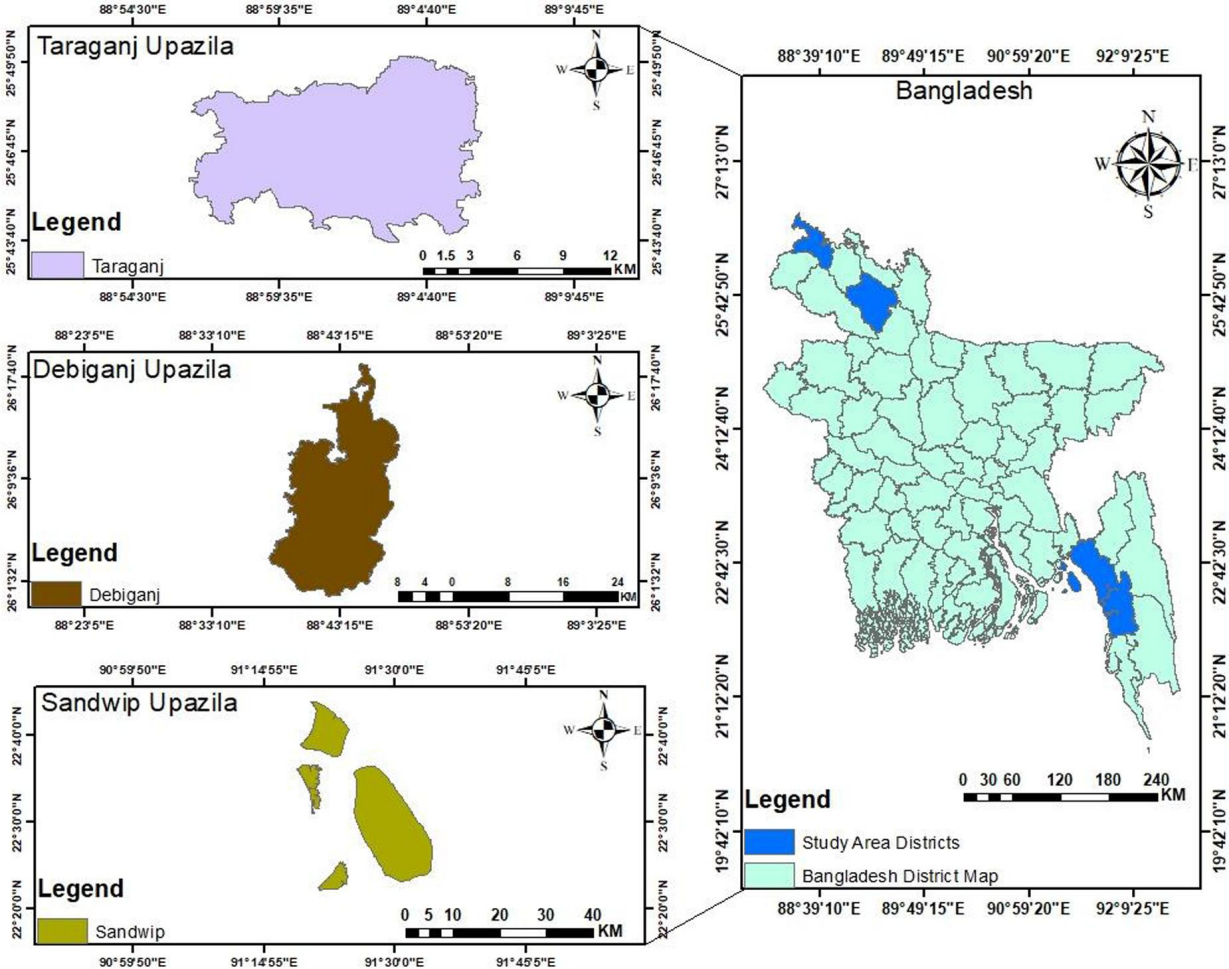
Location of the study area.

A cross-sectional study was conducted from January to March 2024. The research area was divided into two distinct regions: the northern mainland (Taraganj and Debiganj sub-districts) and the offshore islands (Sandwip sub-district) of Bangladesh (Figure 1). Children (particularly 1–4 years old) drowning in ponds has been a significant cause of mortality in this region for generations. Sandwip is an offshore island within Chittagong district and is located in the Bay of Bengal. It is divided into 15 unions. We randomly selected Sarikait and Maitbanga from these unions. Debiganj, a sub-district in the Panchagarh district, is divided into ten unions. Chilahati Union was randomly selected for this survey. There were 7,702 households and a population of 30,100 in Chilahati Union in 2023. In the Rangpur district, Taraganj is a sub-district that comprises five unions. In 2023, with a population of 41,085 and 10,302 households, Hariarkuti Union was also randomly selected for the survey. The two unions had a population of 71,185 and 18,004 households, respectively. We commenced by establishing drowning data from these four unions, namely Sarikait, Maitbanga, Chilahati and Hariarkuti. The dataset includes the age and sex characteristics, time and season, and environmental contextual factors of fatal and non-fatal drownings in the target households over the previous five years and the 12 months preceding this study. The understanding of perceptions regarding childhood drowning will facilitate the development of effective prevention strategies and interventions in these regions and beyond.

### Survey instrument and data collection

A comprehensive review of the literature on existing questionnaires used in comparable studies worldwide was conducted (7, 9, 12, 16–21). The final structured questionnaire was developed after completing a pilot survey and preserving cultural appropriateness using locally understandable terminology, context-specific drowning scenarios, and translation into the native Bengali language to ensure clarity and relevance for the study communities. It was divided into three sections: sociodemographic characteristics, perceptions of childhood drowning, and the childhood drowning death. Deaths caused by drowning in children were assessed using multiple structural options, which included a dichotomous response. The perception section contained a total of 19 semi-structured questions. The questionnaire was available in both the native Bengali and English languages.

Data collection was undertaken by a team of 36 field enumerators, supported by four supervisors responsible for oversight and quality control. All personnel received standardized training covering the study objectives, interview instruments, and ethical considerations. Interviews were conducted with household members who were available and willing to participate at the time of the visit. In households reporting a drowning incident, a purposively selected respondent, preferably the household head or a parent with direct experience of the event, was interviewed. Accordingly, a purposive sampling strategy was adopted to obtain relevant and experience-based information. Households without an available respondent during the initial visit were revisited to maximize participation. Interview excerpts were subsequently translated and transcribed into English by a certified translator in collaboration with a bilingual researcher. In total, 322 individuals participated in the face-to-face survey, including 117 from the Northern Mainland and 205 from Sandwip Island. The unequal sample distribution reflects differences in population distribution, accessibility, and the availability of households reporting childhood drowning experiences across the selected study locations. A comparatively higher number of eligible and accessible households were identified in Sandwip Island during field data collection, resulting in a larger sample from this region.

Several measures were adopted to reduce potential measurement error and social desirability bias during data collection. The questionnaire was pilot-tested prior to the final survey to improve clarity, cultural appropriateness, and consistency of interpretation among respondents. Standardized training was provided to all enumerators to ensure uniform administration of questions and minimize interviewer-related variability. Participants were informed that there were no “right” or “wrong” answers and that all responses would remain anonymous and confidential. Questions related to supervision practices, drowning prevention behaviors, and caregiving responsibilities were phrased neutrally to reduce the likelihood of socially desirable responses.

### Data management and analysis

Statistical analysis was conducted using the “R” program (version 3.6.3; Vienna, Austria) (22). Where necessary, descriptive statistical analyses (frequency and percentage) were conducted. Simple logistic regression analysis was performed to examine the association between each perception-related variable and reported childhood drowning death. Because only one perception-related variable was statistically significant in the simple logistic regression analysis, multiple logistic regression analysis was not conducted.

The binary logistic regression model used in this study can be expressed as follows:

$$\mathrm{logit}(P)\;=\;\ln\;[P/(1-P)]\;=\;\beta_{0}\;+\;\beta_{1}X_{1}$$

where *P* represents the probability of reported childhood drowning death, *β*₀ denotes the intercept, *β*₁ represents the regression coefficient, and X₁ indicates the perception-related predictor variable included in each simple logistic regression model.

Similarly, the multiple linear regression model used to assess factors associated with drowning perception scores can be expressed as:

$$Y=\beta_{0}+\beta_{1}X_{1}+\beta_{2}X_{2}\;+\;\ldots \;\beta_{n}X_{n}+\varepsilon$$

where Y represents the total perception score regarding childhood drowning, *β*₀ is the intercept, *β*₁ to *β*_n_ are regression coefficients, X₁ to X_n_ represent sociodemographic predictor variables, and *ε* denotes the error term. The association between sociodemographic characteristics and total perception score was estimated using simple linear regression analysis and multiple linear regression analysis. Only significant variables with in the simple linear regression analysis were entered into the multiple linear regression model. All statistical analyses were conducted at the 95% confidence interval level.

### Model diagnostics

For the simple logistic regression analysis, model performance was assessed using classification accuracy and ROC curve analysis. Because the final logistic regression model contained only one binary predictor with limited unique fitted probabilities, the Hosmer–Lemeshow goodness-of-fit test was not informative and therefore was not reported.

For the multiple linear regression analysis, multicollinearity was assessed using VIF values. All adjusted GVIF values were below 2, indicating no evidence of serious multicollinearity among predictors. Residual diagnostics were assessed using histogram, Q–Q plot, Shapiro–Wilk normality test, and Breusch–Pagan test. The Shapiro–Wilk test indicated some deviation from normality, while the Breusch–Pagan test suggested mild heteroscedasticity. However, visual inspection of residual plots suggested that these deviations were not severe. Therefore, the model was considered acceptable for interpretation, particularly given the exploratory nature of the study and the moderate sample size. These diagnostic procedures followed standard regression analysis guidance (23).

### Limitations

The findings of this study should be interpreted considering several limitations. First, the use of purposive and non-probability sampling limits the external generalizability of the findings beyond the selected study areas. Second, the cross-sectional design captures associations at a single point in time and does not permit causal inference or assessment of temporal changes. Third, the study relied on retrospective self-reported information regarding drowning experiences and perceptions, which may have introduced recall bias and measurement error. Participants may not have accurately remembered previous drowning incidents, preventive practices, or training experiences over the reference period.

In addition, some responses may have been influenced by social desirability bias, particularly for perception- and behavior-related questions involving child supervision, drowning prevention, and caregiving responsibilities. Respondents may have tended to provide socially acceptable answers rather than fully reflecting their actual practices or beliefs. To minimize these risks, the questionnaire was culturally adapted, pilot-tested, translated into locally understandable Bengali language, and administered through face-to-face interviews by trained enumerators following standardized procedures. Participants were also informed that their responses would remain confidential and anonymous.

Furthermore, the absence of systematically maintained drowning records by relevant government agencies in the study areas limited opportunities to validate reported drowning events using official databases. Despite these limitations, the study provides important exploratory evidence regarding drowning perception and associated sociodemographic factors in underserved mainland and island communities of Bangladesh.

## Results

Childhood drowning remains an important public health concern in Bangladesh, particularly in water-abundant rural and island communities. This study explored community perceptions, preventive practices, and factors associated with childhood drowning using survey data collected from 322 respondents. The findings are presented in three main sections: (1) perception and awareness regarding childhood drowning prevention, (2) perception-related factors associated with reported childhood drowning death, and (3) sociodemographic factors associated with total drowning perception scores.

### Perceptions regarding childhood drowning

Perceptions regarding childhood drowning prevention are presented in Table 1. Overall, awareness regarding the preventability of childhood drowning was high, with 90.37% of respondents believing that child drowning can be prevented. Similarly, 93.48% believed that public awareness and water safety initiatives could reduce drowning incidents, while 98.14% emphasized the importance of awareness-building activities. Additionally, 67.70% of respondents agreed that parental or caregiver negligence contributes to child drowning in their communities.

**Table 1:** Perception related to childhood drowning.

Perception	Frequency (%)
Do you think child drowning is preventable?	
Yes	291 (90.37)
No	31 (9.63)
Do you believe public awareness and water safety focus can reduce child drowning?	
Yes	301 (93.48)
No	21 (6.52)
What would you like to do as a personal initiative to prevent child drowning in your area?	
Installation of barriers at the entrance of reservoirs	182 (56.52)
Conducting awareness activities	101 (31.37)
Establish childcare	39 (12.11)
What are the causes of child drowning in your area?	
A child goes to the reservoir alone	298 (92.55)
Any other reason	12 (3.73)
Inappropriate or dangerous behavior	1 (0.31)
Peer pressure	11 (3.42)
Is it important to raise awareness to reduce child drowning?	
Yes	316 (98.14)
No	6 (1.86)
Do you agree that parental/caregiver negligence is responsible for child drowning in your area?	
Yes	218 (67.70)
No	104 (32.30)
Do you know that you should not jump alone without a rope or a floating item to rescue a drowned person?	
Yes	50 (15.53)
No	272 (84.47)
Do you teach your child (who could understand) what to do if seen someone drowning?	
Yes	24 (5.28)
No	298 (92.55)
Do you know how to conduct Cardiopulmonary Resuscitation (CPR) for a drowned person?	
Yes	17 (5.28)
No	305 (94.72)
If you know, where did you learn it?	
Don't know CPR	305 (94.72)
Family or peers	9 (2.80)
NGO	2 (0.62)
YouTube or any other social media	3 (0.93)
From someone else	3 (0.93)
Will you install a barrier at the entrance to the reservoir to prevent the baby from drowning?	
Yes	285 (88.51)
No	37 (11.49)
Would you consent to any public or private agency installing a barrier at the entrance to your pond or other body of water?	
Yes	313 (97.20)
No	9 (2.80)
Would you send your 1- to 4-year-old child to any government or private childcare center?	
Yes	265 (82.30)
No	57 (17.70)
Would you let your 4- to 5-year-old child participate in any swimming training program organized by any government or private organization?	
Yes	295 (91.61)
No	27 (8.39)
Where did you teach your child to swim?	
Did not learn to swim	114 (35.40)
Pond	198 (61.49)
Any other reservoir	10 (3.11)
Have you received any help or information in the past to prevent child drowning in your area?	
No	322 (100.00)
Do you think you are knowledgeable about water safety and drowning prevention?	
Yes	203 (63.04)
No	119 (36.96)
Have you ever attended a water safety awareness program or workshop?	
Yes	4 (1.24)
No	318 (98.76)
Are you willing to participate in any workshops related to water protection in the future?	
Yes	317 (98.45)
No	5 (1.55)

Despite relatively high awareness levels, important gaps in practical prevention knowledge and skills were identified. A large proportion of respondents (84.47%) reported lacking knowledge regarding appropriate rescue techniques during drowning emergencies, and 94.72% reported not knowing how to perform cardiopulmonary resuscitation (CPR). Only 5.28% reported teaching children how to respond if they witnessed a drowning incident. Furthermore, none of the respondents reported previously receiving formal information or assistance related to childhood drowning prevention.

Respondents generally demonstrated positive attitudes toward community-based prevention strategies. Most participants expressed willingness to install barriers near water bodies (88.51%) and to allow public or private agencies to install protective barriers around ponds or reservoirs (97.20%). Similarly, 82.30% indicated willingness to send children aged 1–4 years to childcare centers, and 91.61% supported participation of children in organized swimming training programs. Additionally, 98.45% expressed willingness to participate in future water safety workshops.

Regarding swimming practices, 35.40% of children had not learned swimming, while 61.49% reportedly learned swimming in ponds, often without structured supervision or formal safety training.

### Perception-related factors

In our ongoing effort to understand childhood drowning, we conducted an analysis of perception-related factors and their association with reported childhood drowning death (Table 2). Table 2 presents the results of the simple logistic regression analysis. No statistically significant association was observed between the perception that parental/caregiver negligence contributes to child drowning and reported childhood drowning death (OR: 0.90, 95% CI: 0.55–1.45, *p* = 0.665). An important finding emerged regarding childcare center attendance. Parents who expressed willingness to send their children aged 1–4 years to childcare centers were associated with a significantly higher likelihood of reported childhood drowning death (OR: 3.69, 95% CI: 2.05–6.80, *p* < 0.001).

**Table 2:** Simple logistic regression analysis of perception-related factors associated with reported childhood drowning death.

Perception-related variables	OR^a^ (95% CI)	*P*-value
Do you know that you should not jump alone without a rope or floating item to rescue a drowned person?		
No	Reference	
Yes	0.96 (0.52, 1.82)	0.907
Do you teach your child what to do if they see someone drowning?		
No	Reference	
Yes	1.48 (0.62, 3.94)	0.396
Do you know how to conduct CPR for a drowned person?		
No	Reference	
Yes	0.65 (0.24, 1.78)	0.394
Do you think child drowning is preventable?		
No	Reference	
Yes	1.44 (0.67, 3.03)	0.341
Do you agree that parental/caregiver negligence is responsible for child drowning in your area?		
No	Reference	
Yes	0.90 (0.55, 1.45)	0.665
Will you install a barrier at the entrance to the reservoir to prevent the baby from drowning?		
No	Reference	
Yes	1.92 (0.96, 3.86)	0.063
What would you like to do as a personal initiative to prevent child drowning in your area?		
Installation of barriers at the entrance of reservoirs	Reference	
Raising awareness, setting up barriers, swimming training programs, and introducing children to water through childcare centers	3.45 (0.66, 25.15)	0.157
Would you send your 1- to 4-year-old child to any government or private childcare center?		
No	Reference	
Yes	3.69 (2.05, 6.80)***	<0.001
Would you consent to any public or private agency installing a barrier at the entrance to your pond or other body of water?		
No	Reference	
Yes	2.15 (0.56, 8.85)	0.260
Would you let your 4- to 5-year-old child participate in any swimming training program organized by any government or private organization?		
No	Reference	
Yes	1.63 (0.73, 3.62)	0.225
How knowledgeable do you think you are about water safety and drowning prevention?		
No	Reference	
Yes	1.14 (0.72, 1.84)	0.575
Have you ever attended a water safety awareness program or workshop?		
No	Reference	
Yes	0.56 (0.03, 4.41)	0.615
Do you believe that public awareness and focus on water safety can reduce child drowning?		
No	Reference	
Yes	2.38 (0.98, 6.01)	0.058
Are you willing to participate in any workshops related to water protection in the future?		
No	Reference	
Yes	2.40 (0.35, 47.33)	0.435

*** *p* < 0.001.

a OR, Odds Ratio; CI, Confidence Interval.

Most other perception-related variables, including knowledge of CPR, awareness of rescue techniques, willingness to install barriers, participation in swimming programs, and attendance at water safety workshops, were not significantly associated with reported childhood drowning death. As only one variable was found to be statistically significant in the simple logistic regression analysis, multiple logistic regression analysis was not conducted. The absence of a significant association between perceived parental negligence and drowning mortality indicates a potential divergence between public perceptions and empirically identified risk factors, which may impede the effectiveness of prevention strategies. The observed positive association between reported willingness to use childcare centers and increased drowning risk is noteworthy. However, this variable reflects stated intention rather than actual utilization of childcare services and therefore may not accurately represent real-world practices.

### Sociodemographic factors

This study also explored how location, income, education, and housing shape people's perceptions regarding childhood drowning. Tables 3, 4 present the results of the simple and multiple linear regression analyses, respectively. Only variables that were statistically significant in the simple linear regression analysis were included in the multiple linear regression model.

**Table 3:** Simple linear regression analysis of sociodemographic characteristics associated with total childhood drowning perception score.

Characteristics	Frequency (%)	*β*^a^ (95% CI)	*P*-value
Area			
Northern mainland	117 (36.34)	Reference	
Sandwip Island	205 (63.66)	−0.04 (−0.06, −0.01)**	0.002
Age (years)			
18–30	71 (22.05)	Reference	
31–45	168 (52.17)	−0.01 (−0.04, 0.02)	0.639
46–50	39 (12.11)	−0.02 (−0.05, 0.01)	0.269
50+	44 (13.66)	−0.01 (−0.06, 0.03)	0.580
Gender			
Female	143 (44.41)	Reference	
Male	179 (55.59)	0.02 (0.00, 0.05)*	0.040
Family type			
Extended	69 (21.43)	Reference	
Single	253 (78.57)	−0.03 (−0.06, 0.00)*	0.027
Number of family members			
1–5	197 (61.18)	Reference	
>5	125 (38.82)	−0.01 (−0.03, 0.01)	0.447
Number of children (<12 years old)			
1–2	241 (74.84)	Reference	
3–4	55 (17.08)	−0.03 (−0.06, 0.00)*	0.043
>4	26 (8.07)	−0.02 (−0.06, 0.02)	0.419
Education			
Illiterate	81 (25.16)	Reference	
Primary	87 (27.02)	−0.02 (−0.05, 0.01)	0.176
Secondary or more	154 (47.83)	0.03 (0.01, 0.06)*	0.019
Occupation			
Farmers or fishers	72 (22.36)	Reference	
Small business	48 (14.91)	0.01 (−0.01, 0.04)	0.329
Miscellaneous	186 (57.76)	0.02 (−0.02, 0.05)	0.438
Unemployed	16 (4.97)	0.01 (−0.05, 0.06)	0.857
Income of household (BDT^b^)			
<10,001	204 (63.35)	Reference	
10,001–20,000	84 (26.09)	0.04 (0.02, 0.07)***	0.001
>20,000	34 (10.56)	0.08 (0.05, 0.12)***	<0.001
Residence type			
Kacha (muddy)	230 (71.43)	Reference	
Pucca (Building)	30 (9.32)	0.05 (0.01, 0.08)*	0.016
Semi-pucca	62 (19.25)	0.06 (0.03, 0.09)***	<0.001
Number of households living and sharing certain area			
1–3	151 (46.89)	Reference	
>3	171 (53.11)	−0.04 (−0.06, −0.02)***	0.001

* *p* < 0.05;.

** *p* < 0.01;.

*** *p* < 0.001.

a β, regression coefficient; CI, Confidence Interval; BDT^b^, Bangladeshi Taka (1 USD = about 120 BDT).

The outcome variable was total childhood drowning perception score. Confidence intervals are presented as lower and upper limits (24).

**Table 4:** Multiple linear regression analysis of sociodemographic characteristics associated with total childhood drowning perception score.

Variables	β^a^ (95% CI)	*P*-value
Area		
Northern mainland	Reference	
Sandwip Island	−0.04 (−0.06, −0.01)**	0.007
Gender		
Female	Reference	
Male	−0.00 (−0.03, 0.02)	0.824
Family type		
Extended	Reference	
Single	−0.03 (−0.06, −0.00)*	0.030
Education		
Illiterate	Reference	
Primary	−0.01 (−0.04, 0.02)	0.414
Secondary or more	0.03 (−0.00, 0.05)	0.061
Income of household (BDT^b^)		
<10,001	Reference	
10,001–20,000	0.04 (0.01, 0.06)*	0.014
>20,000	0.08 (0.04, 0.12)***	<0.001
Residence type		
Kacha (muddy)	Reference	
Pucca (Building)	0.01 (−0.02, 0.04)	0.569
Semi-pucca	0.03 (0.00, 0.06)*	0.029
Number of households living and sharing certain area		
1–3	Reference	
>3	−0.03 (−0.05, −0.00)*	0.034
Number of children (<12 years old)		
1–2	Reference	
3–4	−0.03 (−0.05, 0.00)	0.069
>4	0.01 (−0.03, 0.04)	0.769

* *p* < 0.05.

** *p* < 0.01.

*** *p* < 0.001.

a β, regression coefficient; CI, Confidence Interval; BDT^b^, Bangladeshi Taka (1 USD = about 120 BDT).

The outcome variable was total childhood drowning perception score. Confidence intervals are presented as lower and upper limits (24).

Intriguingly, residing on Sandwip Island was associated with a slightly lower perception score regarding childhood drowning risk in both the simple linear regression analysis (*β* = −0.04, 95% CI: −0.06 to −0.01, *p* = 0.002) and the multiple linear regression analysis (*β* = −0.04, 95% CI: −0.06 to −0.01, *p* = 0.007). The findings also demonstrated increasing awareness with rising household income. Respondents from households earning 10,001–20,000 BDT and >20,000 BDT showed significantly greater perception scores regarding childhood drowning risk compared to those earning <10,001 BDT. In the multiple linear regression analysis, respondents from households earning >20,000 BDT had the strongest positive association with perception scores (*β* = 0.08, 95% CI: 0.04 to 0.12, *p* < 0.001).

Male respondents, individuals living in single-family households, respondents living in semi-pucca houses, and households with more than three families sharing the same area also showed significant associations in the simple linear regression analysis. However, some of these associations lost statistical significance after adjustment in the multiple linear regression model. Although the findings suggested a trend toward higher perception scores among respondents with secondary or higher education, the association did not remain statistically significant in the adjusted model.

No statistically significant associations were observed between age groups, occupation, household size, or number of children and childhood drowning perception scores in the adjusted analysis. Overall, geographic location and household income emerged as the most consistent factors associated with perception regarding childhood drowning risk.

## Discussion

This study examined perception-related factors associated with childhood drowning and explored how sociodemographic characteristics influence awareness regarding drowning risk in Bangladesh. The findings reveal important gaps between general awareness and practical preventive knowledge, while also highlighting the influence of geography and socioeconomic status on drowning perception. This discussion analyzes the findings, compares them with evidence from Bangladesh and other low- and middle-income countries where childhood drowning remains a major public health concern, and explores potential solutions within Bangladesh's unique cultural and socioeconomic context.

The study reveals a paradox; while 90.37% of respondents believe child drowning is preventable, practical prevention measures are largely absent. This disconnect between awareness and action is further highlighted by the fact that 67.70% of respondents acknowledge parental or caregiver negligence as a contributing factor to drowning incidents. Thus, despite high awareness of drowning risks, many parents lack specific knowledge about prevention strategies. This knowledge gap underscores the need for community-based programs and low-cost solutions to enhance awareness and equip parents with the necessary tools to prevent drowning incidents (25).

A pilot drowning prevention in Bangladesh suggests that the “creches” intervention implemented across 7 sub-districts reduced drowning rates among children under 5 by 88% (26). Another pilot project in Thailand suggests that community-based interventions, including improved supervision and environmental modifications, can significantly reduce childhood drowning rates (27). Our study suggests that a large proportion of respondents do not know how to perform CPR on a drowning victim, indicating a critical gap in life-saving skills. Knowing CPR is crucial in preventing child drowning deaths, as it can significantly increase survival rates in emergencies. Drowning is often a hypoxic event, where a lack of oxygen leads to cardiac arrest. Immediate CPR, especially with rescue breaths, helps restore oxygen flow to the brain and vital organs, potentially saving the child's life. Community-based CPR training is essential to ensure more people are prepared to respond to drowning incidents (28).

100% of respondents report having never received any help or information on child drowning prevention, pointing to a severe lack of public education initiatives. These findings echo challenges faced in other low- and middle-income countries. A systematic review of drowning prevention found that while swimming skills are crucial, they must be complemented by water safety education and improved supervision (13). Additionally, a Bangladeshi study found that there was a higher risk of childhood drowning in homes with illiterate parents (7). It also fits with global developments in child safety, where parental education is frequently associated with children's healthier outcomes (29).

Our study's findings underscore the urgent need for comprehensive water safety education. Knowledge gaps have been observed in other South Asian countries, such as Sri Lanka, where a study in rural areas found low water safety knowledge levels among children and adults (30).

The findings of this study should also be interpreted within the broader context of household drowning risks. While ponds, rivers, and open water bodies remain major drowning locations in Bangladesh, global evidence suggests that infants and toddlers may also drown in domestic environments, including swimming pools, bathtubs, buckets, and other water storage containers when left unsupervised, even for short periods (1, 12). In many households, particularly in low-resource settings, buckets and containers used for storing water for daily household activities may unintentionally become hazardous for young children. This highlights that drowning prevention should not be limited only to outdoor environmental interventions but should also incorporate household water safety practices, safe water storage, restricted access to bathrooms or water containers, and continuous adult supervision of infants and toddlers.

Based on the study findings and considering the economic constraints, a multi-faceted approach is recommended. Firstly, we recommend implementing safe and structured swimming education programs. We further recommend launching widespread CPR and first aid training initiatives to address the critical knowledge gap in life-saving skills. Additionally, leveraging high community willingness to install barriers at local water bodies, focusing on high-risk areas, is crucial. Lastly, expanding childcare center initiatives, would capitalize on the 82.30% willingness to send young children to these centers. In February 2022, the Government of Bangladesh approved a $32 million, three-year program to reduce drowning among children throughout the country (31). As part of the program, the government will take over the 2,500 childcare sites established and funded by Bloomberg Philanthropies since 2012 and will expand the program by adding an additional 5,500 childcare sites to provide supervision to 200,000 children ages 1–5. Bloomberg Philanthropies will provide technical assistance to the government in the scale up and implementation of the 8,000 childcare sites.

One might assume that parents who perceive themselves as negligent would be more likely to experience a drowning tragedy. However, our study reveals that there is no significant association between the perception of parental negligence and childhood drowning deaths. This finding challenges the common narrative that blames parents for these incidents and suggests that the roots of this issue run deeper than individual responsibility.

Our research uncovered a correlation that requires careful interpretation. Parents who were willing to send their 1- to 4-year-old children to childcare centers showed a statistically significant association with reported childhood drowning deaths. This association should not be interpreted as childcare increasing drowning risk, as supervised childcare is an established and evidence-based drowning prevention measure. In the Bangladeshi context, willingness to use childcare centers may also reflect the realities of working-class and middle-income households, where parents or caregivers often spend long hours away from home for employment or livelihood activities. Such families may actively seek childcare support because continuous supervision of young children becomes challenging during working hours. In addition, households previously exposed to drowning incidents may become more aware of supervision needs and therefore more willing to adopt childcare services as a protective strategy. Rather than questioning the effectiveness of childcare itself, this finding highlights the need to ensure consistent quality, proper supervision, and safety standards in all childcare settings, particularly as Bangladesh continues expanding community-based childcare programs. Global evidence, including WHO recommendations, clearly establishes childcare and supervised group care as effective drowning-prevention strategies (32, 33).

The study paints a vivid picture of how geography shapes perception. Residents of Sandwip Island showed a slightly lower perception of childhood drowning risk compared to their mainland counterparts. This statistically significant difference hints at the complex interplay between environment, culture, and risk awareness. By conducting comparative analyses across diverse regions, we gain a deeper understanding of the complex factors influencing childhood drowning risks. This knowledge can inform the development of more comprehensive and evidence-based policies and programs to address this significant public health issue. While the northern mainland in Bangladesh serves as our baseline for typical drowning risks, the island of Sandwip in the Bay of Bengal tells a different story. This discrepancy poses interesting queries. The number of children drowning in island areas is startlingly high. With almost 74,000 drowning deaths in 2019, the Western Pacific Region likewise records a high number of drowning deaths. This region's top cause of mortality for children under five is drowning, highlighting how vulnerable young children are in island and coastal communities (34). In other research, geographic differences have also been noted. For example, the prevalence of open water bodies like ponds, rivers, and ditches that are frequently unprotected and easily accessible to children, raises the risk of childhood drowning in Bangladesh's rural areas (9). On the other hand, cities may have greater infrastructure and security systems, which lowers the chance of an unintentional drowning. There is an increased risk of drowning among children in areas that flood frequently. Floods produce transient bodies of water and raise the environmental water hazard, which increases the risk of drowning (1). Thus, residents of Sandwip should have better perceptions regarding childhood drowning. This may offer opportunities for further study.

From the findings, a compelling narrative emerges—one where perception seems to flow with current household income. Those in higher income brackets show more awareness of childhood drowning risks. This finding underscores the need for targeted awareness campaigns that bridge the gap between income groups, ensuring that life-saving information reaches every household, regardless of economic status. One report emphasizes the critical need to address socioeconomic disparities when developing drowning prevention strategies (35). It discusses how social and economic factors, including income, education, and access to water safety resources, are key determinants of drowning risk. The report highlights that interventions must be tailored to meet the needs of different communities, particularly those in low- and middle-income areas.

Our study population was concerned about barriers to open water. One of the six main causes of drowning, according to the World Health Organization, is the absence of physical barriers between people and water. Without these barriers, children can easily access dangerous water areas, which increases the risk of drowning (16). Unrestricted access to irrigation canals, wells, and ponds raises children's drowning risk (9). Environmental hazards, such as unobstructed, open water sources, are prevalent in countries with low or middle incomes. Due to the increased likelihood of children coming into contact with hazardous water bodies while going about their regular lives, these risks raise the chance of juvenile drowning (9). Activities to raise community awareness and enhance safety must address the lack of barriers (17).

The expansion of Integrated Community-Based Childcare (ICBC) to underserved regions in Bangladesh is essential. Given the success of the ICBC model, policymakers and the government should prioritize scaling this project further, ensuring comprehensive coverage that includes all high-risk areas across the country. This is a call to action for an inclusive national strategy that safeguards every child from preventable drowning deaths.

## Conclusions and recommendations

Our study highlights the complexities surrounding childhood drowning in Bangladesh, identifying critical gaps between parental perceptions and actual risks. The disconnect between perceived parental negligence and drowning incidents suggests a lack of public understanding, which could undermine prevention efforts. Although our analysis showed a statistical association between willingness to send children to childcare centers and reported drowning deaths, this finding should not be interpreted as childcare increasing drowning risk. The observed association in our sample likely reflects contextual or unmeasured household factors rather than the impact of childcare itself. Furthermore, socioeconomic factors play a significant role in shaping drowning awareness, with higher-income families exhibiting greater understanding of the risks. This underscores the need for tailored strategies that address the specific challenges faced by lower-income communities.

To address these issues, we recommend the following targeted interventions:

**Public Awareness Campaigns**: Develop community-specific education programs to address the gap between perceived and actual drowning risks. These campaigns should emphasize constant supervision near both open and household water sources, practical prevention techniques, safe water storage practices, and restricting young children's unsupervised access to bathrooms, buckets, ponds, and other hazardous water environments, utilizing local media and community leaders for broader outreach.

**Childcare Center Safety Audits**: Conduct immediate safety audits of existing childcare facilities and enforce strict water safety protocols, staff training, and regular monitoring to mitigate the risks associated with childcare attendance.

**Income-Sensitive Interventions**: Introduce subsidized swimming lessons and water safety equipment for lower-income communities. Partner with local businesses to support community water safety initiatives, ensuring that all economic groups have access to necessary resources.

**Swimming and Water Safety Education**: Establish community-based swimming programs for children aged 4–5 and integrate water safety education into school curricula. Train local volunteers as instructors to sustain these programs over time. Children should also be encouraged to participate in age-appropriate swimming and survival floating lessons from early childhood under trained supervision, as swimming competency is considered an important component of drowning prevention globally.

## Data Availability

The raw data supporting the conclusions of this article will be made available by the authors, without undue reservation.

## References

[1] WHO. Drowning [WWW Document] (2023). Available online at: https://www.who.int/news-room/fact-sheets/detail/drowning (Accessed 7.27.24).

[2] Cenderadewi M Franklin RC Devine S. Socio-ecological nature of drowning in low-and middle-income countries: a review to inform health promotion approaches. Int J Aquat Res Educ. (2020) 12:6. 10.25035/ijare.12.02.06

[3] Franklin RC Peden AE Hamilton EB Bisignano C Castle CD Dingels ZV. The burden of unintentional drowning: global, regional and national estimates of mortality from the global burden of disease 2017 study. Inj Prev. (2020) 26:i83–95. 10.1136/injuryprev-2019-043484 32079663 PMC7571364

[4] Rahman A Peden AE Ashraf L Ryan D Bhuiyan AA Beerman S. Drowning: global burden, risk factors, and prevention strategies. In: Mequeen DV, editor. Oxford Research Encyclopedia of Global Public Health. Oxford, UK. (2021) 197.

[5] Scarr JP Buse K Norton R Meddings DR Jagnoor J. Tracing the emergence of drowning prevention on the global health and development agenda: a policy analysis. Lancet Glob Health. (2022) 10:e1058–66. 10.1016/S2214-109X(22)00074-2 35461520 PMC9197772

[6] Biswas A Hossain J Abdullah A Dalal K Mashreky S Hawlader D. Rescue and emergency management of a water-related disaster: a Bangladeshi experience. Asian J Med Health. (2017) 4:1–9. 10.9734/AJMAH/2017/32163

[7] Hossain M Mani KKC Sidik SM Hayati KS Rahman AKMF. Socio-demographic, environmental and caring risk factors for childhood drowning deaths in Bangladesh. BMC Pediatr. (2015) 15:1–6. 10.1186/s12887-015-0431-7 26357879 PMC4566200

[8] Hossain MJ Al-Mamun M Alam M Khatun MR Sarker MMR Islam MR. Child drownings in Bangladesh: need for action. BMJ Paediatrics Open. (2022) 6:e001464. 10.1136/bmjpo-2022-001464 36053622 PMC9171250

[9] Rahman A Alonge O Bhuiyan A-A Agrawal P Salam SS Talab A. Epidemiology of drowning in Bangladesh: an update. Int J Environ Res Public Health. (2017) 14:488. 10.3390/ijerph14050488 28475138 PMC5451939

[10] Lukaszyk C Mittal S Gupta M Das R Ivers R Jagnoor J. The impact and understanding of childhood drowning by a community in West Bengal, India, and the suggested preventive measures. Acta Paediatr. (2019) 108:731–9. 10.1111/apa.14592 30252948

[11] Alam E Hattawi KSA Akter H Alam J Alvarez E Sufi F. Socioeconomic, Demographic and Environmental Factors of Child Drownings in Northern Bangladesh. London: Injury Prevention (2025). 10.1136/ip-2024-045434 PMC13638329 39890442

[12] Al-Mamun M Alam M Hossain MJ Khatun MR Das PK Alam F. Child drowning and associated risk factors: findings from a qualitative study in Bangladesh. Health Sci Rep. (2023) 6:e1380. 10.1002/hsr2.1380 37396561 PMC10308348

[13] Wallis BA Watt K Franklin RC Taylor M Nixon JW Kimble RM. Interventions associated with drowning prevention in children and adolescents: systematic literature review. Inj Prev. (2015) 21:195–204. 10.1136/injuryprev-2014-041216 25189166

[14] WHO. Global status Report on Drowning Prevention 2024. Geneva: World Health Organization (2024).

[15] Hyder AA Alonge O He S Wadhwaniya S Rahman F Rahman A. Saving of children’s lives from drowning project in Bangladesh. Am J Prev Med. (2014) 47:842–5. 10.1016/j.amepre.2014.07.050 25241200

[16] Alam E. A neglected national crisis [WWW Document]. The Daily Star (2023). Available online at: https://www.thedailystar.net/opinion/views/news/neglected-national-crisis-3377411 (Accessed 7.27.24).

[17] Button C Ng JL Burnay C van Duijn T. Application of ecological dynamics principles to drowning prevention. Asian J Sport Exerc Psychol. (2022) 2:59–66. 10.1016/j.ajsep.2022.04.001

[18] Kiakalayeh AD Mohammadi R Ekman DS Chabok SY Janson B. Unintentional drowning in northern Iran: a population-based study. Accid Anal Prev. (2008) 40:1977–81. 10.1016/j.aap.2008.08.008 19068303

[19] Liu Z Kong F Yin L Wang A Xiong L Xie D. Epidemiological characteristics and influencing factors of fatal drowning in children under 5 years old in hunan province, China: case-control study. BMC Public Health. (2019) 19:955. 10.1186/s12889-019-7241-z 31315598 PMC6637556

[20] Wang M Liu Y Kang L He C Miao L Huang J. Social and environmental risk factors for the accidental drowning of children under five in China. BMC Public Health. (2020) 20:1553. 10.1186/s12889-020-09650-0 33059656 PMC7559335

[21] WHO. Global report on drowning: preventing a leading killer (2014).

[22] R Development Core Team. R: A Language and Environment for Statistical Computing, Version 4.2.2. R Foundation for Statistical Computing. Vienna, Austria: WWW Document (2022).

[23] Field A Field Z Miles J. Discovering statistics using R (2012).

[24] Zafri NM Prithul AA Baral I Rahman M. Exploring the factors influencing pedestrian-vehicle crash severity in Dhaka, Bangladesh. Int J Inj Contr Saf Promot. (2020) 27:300–7. 10.1080/17457300.2020.1774618 32498599

[25] Hossain MM. World Drowning Prevention Day: Bangladesh’s Public Health Diplomacy at the UN. Dhaka: Prothomalo (2023).

[26] Alonge O Bishai D Wadhwaniya S Agrawal P Rahman A Dewan Hoque EM. Large-scale evaluation of interventions designed to reduce childhood drownings in rural Bangladesh: a before and after cohort study. Inj. Epidemiol. (2020) 7:17. 10.1186/s40621-020-00245-2 32389128 PMC7212604

[27] Sansiritaweesook G Kanato M. Development of the Model for Local Drowning Surveillance System in Northeastern Thailand 98 (2015).

[28] Moran K Stanley T Rutherford A. Toddler drowning prevention: teaching parents about child CPR in conjunction with their child’s in-water lessons. Int J Aquat Res Educ. (2012) 6:254–56. 10.25035/ijare.06.04.06 17345725

[29] Gakidou E Cowling K Lozano R Murray CJ. Increased educational attainment and its effect on child mortality in 175 countries between 1970 and 2009: a systematic analysis. Lancet. (2010) 376:959–74. 10.1016/S0140-6736(10)61257-3 20851260

[30] Ekanayaka J Geok CK Matthews B Dharmaratne SD. Influence of a survival swimming training programme on water safety knowledge, attitudes and skills: a randomized controlled trial among young adults in Sri Lanka. Int J Environ Res Public Health. (2021) 18:11428. 10.3390/ijerph182111428 34769944 PMC8583420

[31] Bloomberg Philanthropies. Preventing Drowning. New York: Bloomberg Philanthropies (n.d).

[32] Vecino-Ortiz AI Jafri A Hyder AA. Effective interventions for unintentional injuries: a systematic review and mortality impact assessment among the poorest billion. Lancet Glob Health. (2018) 6:e523–34. 10.1016/S2214-109X(18)30107-4 29653626

[33] WHO. Preventing Drowning: An Implementation Guide. Geneva: World Health Organization (2017).

[34] WHO. Drowning in the Western Pacific [WWW Document] (2024). Available online at: https://www.who.int/westernpacific/health-topics/drowning (Accessed 7.29.24).

[35] World Health Organization. Global Report on Drowning: Preventing a Leading Killer. Geneva: World Health Organization (2014).

[36] WMA. WMA - The World Medical Association-WMA Declaration of Helsinki – Ethical Principles for Medical Research Involving Human Subjects (n.d).

